# Extension of Maximal Lifespan and High Bone Marrow Chimerism After Nonmyeloablative Syngeneic Transplantation of Bone Marrow From Young to Old Mice

**DOI:** 10.3389/fgene.2019.00310

**Published:** 2019-04-12

**Authors:** Marina V. Kovina, Alexey V. Karnaukhov, Mikhail E. Krasheninnikov, Artem L. Kovin, Sarul T. Gazheev, Larisa A. Sergievich, Elena V. Karnaukhova, Elena V. Bogdanenko, Maxim V. Balyasin, Yury M. Khodarovich, Tatyana G. Dyuzheva, Alexey V. Lyundup

**Affiliations:** ^1^Institute for Regenerative Medicine, Sechenov University, Moscow, Russia; ^2^Institute of Cell Biophysics, Russian Academy of Sciences, Pushchino, Russia; ^3^Institute of General Pathology and Pathophysiology, Russian Academy of Sciences, Moscow, Russia; ^4^Department of Molecular Immunology, Shemyakin-Ovchinnikov Institute of Bioorganic Chemistry, Russian Academy of Sciences, Moscow, Russia; ^5^Department of Hospital Surgery, Sechenov University, Moscow, Russia

**Keywords:** bone marrow transplantation, mesenchymal stem cells, longevity, life extension, cryobank

## Abstract

The goal of this work was to determine the effect of nonablative syngeneic transplantation of young bone marrow (BM) to laboratory animals (mice) of advanced age upon maximum duration of their lifespan. To do this, transplantation of 100 million nucleated cells from BM of young syngeneic donors to an old nonablated animal was performed at the time when half of the population had already died. As a result, the maximum lifespan (MLS) increased by 28 ± 5%, and the survival time from the beginning of the experiment increased 2.8 ± 0.3-fold. The chimerism of the BM 6 months after the transplantation was 28%.

## Introduction

Increasein maximum lifespan (MLS) is the most significant indicator of hitting the basic mechanisms of aging, in particular, regarding age-related loss of stem cells (Colvin et al., 2004) and cell damage accumulation (Kujoth et al., 2005; Baar et al., 2017). Self-renewal of tissues occurs continuously: thus, in the heart of rats, about 7% of the cells are replaced yearly (Cheng et al., 1996), while the renewal of blood and epithelial tissues is much faster. It is believed that tissue renewal involves special resident stem cells. The participation of circulating stem cells in tissue renewal is poorly studied. Our *in vitro* studies showed that under certain conditions undifferentiated stem cells can effectively differentiate into the cell type to which their cellular microenvironment belongs (Kovina and Khodarovich, 2011), which supports the possibility of tissue renewal on intravenous administration of stem cells (SCs). This could explain the effective healing by bone marrow (BM) transplantation of not only blood diseases, but also of systemic diseases such as mucopolysaccharidosis, senile hearing loss, and bullous epidermolysis (Birkenmeier et al., 1991; Iwai et al., 2001; Corti et al., 2004; Willenbring et al., 2004; Wagner et al., 2010). We and other authors have shown earlier that radiation-free BM transplantation slows aging (Kamminga et al., 2005; Li et al., 2010; Shen et al., 2011; Kovina et al., 2013; Karnaukhov et al., 2015). Our approach differs from studies of other researchers by the combination of the following parameters (i) larger amount of transplanted material, (ii) close relation of donors and recipients, and (iii) absence of radiation and chemotherapy toxic to the body. Given future clinical application, it is desirable to develop this technique in older animals, because at an earlier age, when the body has a sufficiently large stock of stem cells, the effectiveness of other methods of cell damage elimination and life prolongation is high (Spaulding et al., 1997; Aon et al., 2016), and the risk of invasive interference is not great.

The purpose of this work was to determine the effect on the MLS of transplantation initiated at the age when half of the population has already died of old age. By this age, the content of SCs in the BM falls by more than 10 times, and substitution by the transplanted material can occur without the myeloablative conditioning of the recipients. In the current work, as compared to our previous study, BM donors were produced within the same small-sized stock of B10-GFP mice and were differing from the recipients only by the presence of a green fluorescent protein (GFP) transgene (heterozygous strain). Also, there were more mice in the groups. This allowed us to increase the efficiency of transplantation, to show its effect on the MLS, and to increase statistical reliability of the result. In the end, we discuss ways to solve the issue of donation and SC sources, which is very urgent now and might become even more complicated in the future, especially in view of geriatric application of stem cells.

## Materials and Methods

### Animals

The animal study was carried out in accordance with the recommendations of the local ethical committee of Sechenov First Moscow State Medical University and the study protocol was approved by the local ethical committee of Sechenov First Moscow State Medical University (protocol 05-14). Two groups of recipient mice were used for the experiments. All the recipients were GFP-negative mice of B10-GFP line, which is heterozygous for the GFP transgene. Originally there were 20 animals in the first control group. In the second, experimental group, there were 56 animals, five of which were used to control the level of chimerism and were excluded from mortality statistics. Beginning from the age of 15 months, when about 50% of the mice remain alive, the experimental animals received a series of intravenous bone marrow (BM) injections from young donors. Mice aged 3–15 weeks and heterozygous for the green fluorescent protein transgene (i.e., expressing GFP) of the same B10-GFP line were used as BM donors. All mice were purchased from the same nursery where this mouse line was kept as a small herd, thereby increasing their inbredness and also reducing their average lifespan.

### Isolation and Transplantation

Isolation and transplantation of BM were carried out according to the procedure described by Colvin et al. (2004) with minor modifications. The animals were sacrificed by cervical dislocation and sterilized for 3–5 min in 70% ethanol. The bones of the entire skeleton (scull, spine, and femurs) were cleaned of muscles and crushed in a sterile mortar with a pestle (ethanol- and UV-treated) in 5 ml of sterile Hank's Balanced Salt Solution (HBSS) (Life Technologies Gibco BRL) with 10 U/ml heparin. The resulting suspension was filtered through a 70 μm filter (BD Biosciences), the filter was washed with 2 ml of the buffer, and the two filtrates were pooled and centrifuged for 5 min at 340 g. The pellet was resuspended in 8 ml of HBSS/heparin and centrifuged for 5 min at 340 g. The pellet was resuspended in same buffer (total volume 2–4 ml), and the cells were counted in a Goryaev chamber or with an automatic cell counter. The usual yield was 2–4 × 10^8^ nucleated cells (5–6 × 10^7^ nucleated cells per ml) depending on the donor's age. Immediately before injection (within 20 min), the cells were additionally filtered through a 40 μm filter (BD Biosciences).

A total of 15–20 × 10^6^ cells per recipient animal were slowly, during about 1 min, injected in a volume of 200–300 μl per animal with an insulin syringe (0.33GX12.7 mm needle) via the tail vein; the tail was prewarmed for 2–3 min in a 44°C water bath. The animal to be injected was restrained inside a plastic bottle with the tail coming out through a perforated lid, and clipped during the needle penetration into the vein; the clip was removed when the needle was inserted and injection begun. Up to 15 animals were transplanted during each transplantation day. Transplantations were repeated 6 times within 3 months, with 10–20-day intervals.

### Chimerism Estimation

To monitor the level of chimerism (degree of engraftment of donor cells), the fraction of GFP^+^ cells in the recipients' cell material was assayed. To do this, 200 μl of cell suspension at 10–20 million cells per ml was placed onto a slide, 10 μl of the NucBlue (Thermo Scientific) solution was added, and after incubation for 5 min the cells were covered with a cover glass. Images were obtained by taking 10–20 photographs evenly distributed over the entire preparation, with excitation wavelengths for NucBlue (Hoechst 33342) and GFP using Cellinsight CX7 (Thermo Scientific). The mean percentages of fluorescent cells were determined automatically by the software. To assay fluorescing structures in solid tissue, a piece of tissue of ~1 mm^3^ volume was squashed between the slide and the cover glass and pictures were taken on a NICON 2000 fluorescence microscope.

### Statistical Treatment of the Data

The maximum lifespan for the experimental group of animals (MLS^exp^) was defined as the average lifespan of 10% of the most long-lived mice. The MLS for the control mice (MLS^C^) was determined in two ways. The first value, ${\text{MLS}}_{1}^{\text{C}}$, was a direct estimate of average lifespan of 10% of most long-lived animals from the first group of mice (i.e., 2 out of 20), and the second, ${\text{MLS}}_{2}^{\text{C}}$ was calculated using approximation of the survival curve of the experimental group of mice that died from natural causes before BM transplantations began. The approximation was done by using the nonlinear approximation function from NonlinearModelFit of the MATHEMATICA software package. The confidence interval for *P* = 0.05 was calculated in the standard way using the Student criterion. The two values (${\text{MLS}}_{1}^{\text{C}}$ and ${\text{MLS}}_{2}^{\text{C}}$) thus obtained were compared, and the resulting MLS^C^ value was calculated. This method allowed us to increase the precision of the MLS^C^ calculation, since the number of animals was limited.

## Results

**Figure 1:**
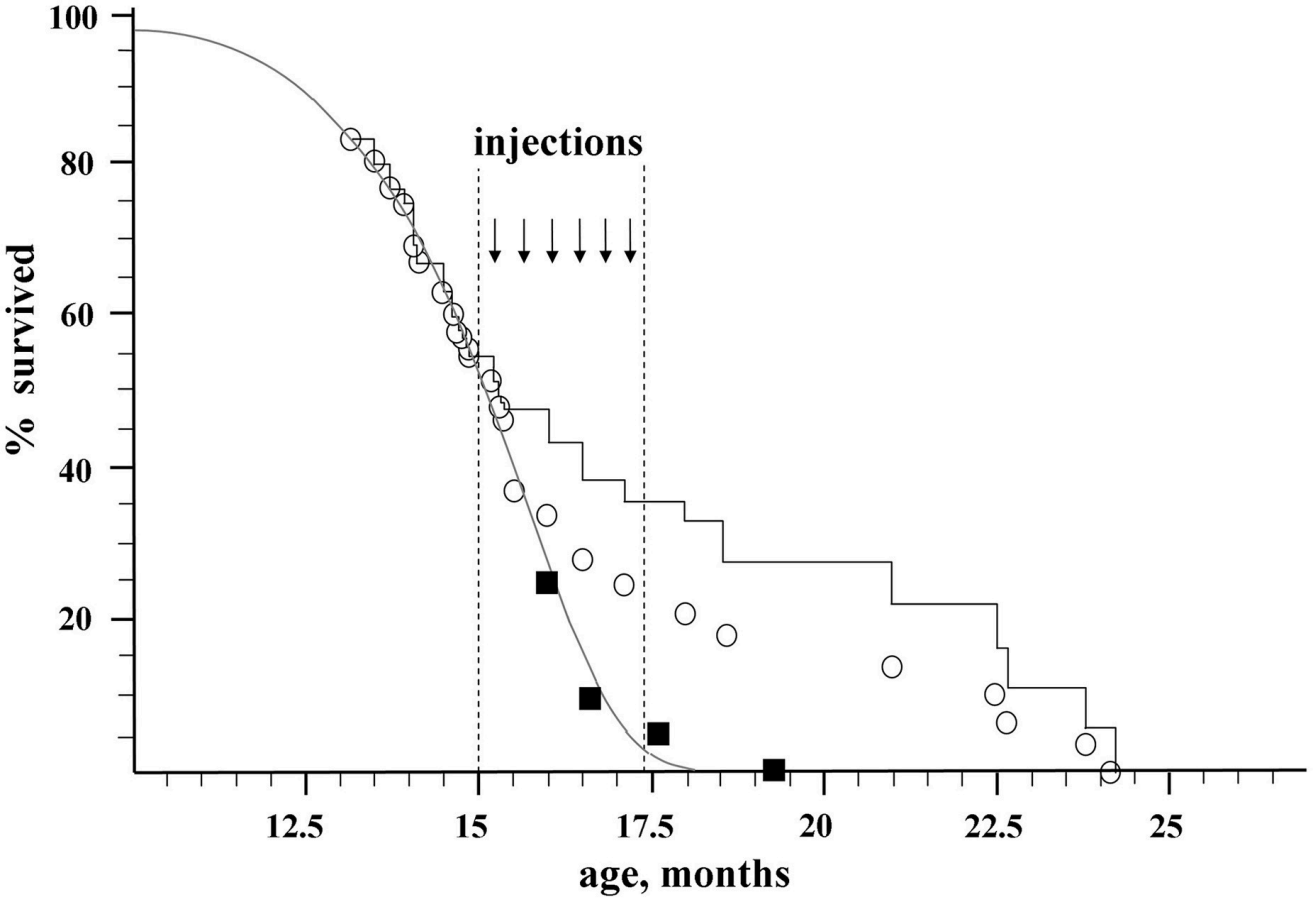
Effect of nonablative transplantation of syngeneic BM of young donors on the population dynamics of aging recipients. Open circles, experimental group; black squares, control group; gray curve, Gompertz–Makeham curve of control group; black stepped curve, experimental group corrected for embolism (embolic animals excluded).

Two groups of recipient mice were used for the experiments. All the recipients were GFP-negative mice of B10-GFP line, which is heterozygous for the GFP transgene. In the first, control group, there were originally 20 animals. In the second, experimental group, there were 56 animals. Beginning from the age of 15 months, when about 50% of the mice remained alive, the experimental animals received a series of intravenous injections of BM from young (3–15 weeks-old) donors, which were GFP positive individuals of the same B10-GFP line. Figure 1 shows the effect of syngeneic transplantation of BM of young donors on the lifespan of the recipients.

The control group was used to directly determine the maximum lifespan of untreated mice (${\text{MLS}}_{1}^{C}$). The number of animals in the control group vs. their age is shown by black squares in Figure 1. The ages of death of the last 10% of this group (two mice) were 17.6 and 19.3 months, which gives a direct estimate for the control group of mice: ${\text{MLS}}_{1}^{C}$ = 18.45 ± 2.6 months. This value of ${\text{MLS}}_{1}^{C}$ has a high standard deviation due to a rather small size of the control group. In order to improve precision of MLS^*C*^ we used data on mortality in the experimental group before transplantation (e.g., before the age of 15 months) when the mortality was due to natural causes. Assuming the standard form of the survival curve in accordance with the Gompertz–Makeham law (Makeham, 1860)

$$\begin{array}{l} f\left(t\right)=N_{0}\cdot Exp\left(-\frac{Exp\left(\mu_{0}+\mu_{1}t\right)}{\mu_{1}}\right)\;, \end{array}$$

where

$$\begin{array}{l} N_{0}=100\%\cdot Exp\left(-\frac{Exp\left(\mu_{0}\right)}{\mu_{1}}\right),\mu_{1}\text{ and }\mu_{2}-\text{parameters} \end{array}$$

and based on the requirement to minimize the quadratic deviation of the model curve from the natural loss of animals in the experimental group (open circles, before transplantation), the numerical values of the parameters were determined as follows: μ_0_ = −11.4 and μ_1_ = 0.7.

This makes it possible to calculate the maximum lifespan, i.e., the average lifespan of animals who lived from t_10_ to **∞**, for the mice in the experimental group before treatment (transplantation), which thus serves as an additional control (${\text{MLS}}_{2}^{C}$):

$$\begin{array}{l} {\text{MLS}}_{\text{2}}^{C}=\frac{\overset{\infty }{\underset{t_{10}}{\int }}t\cdot f\left(t\right)dt}{\overset{\infty }{\underset{t_{10}}{\int }}f\left(t\right)dt}=17.2\pm 0.6\text{ months}, \end{array}$$

where *t*_10_ is the time point when there were 10% of the animals left in the population.

The ${\text{MLS}}_{2}^{\text{C}}$ value is in a good agreement with ${\text{MLS}}_{1}^{\text{C}}$ obtained with the use of first method of estimating the MLS in the control group. The relatively small difference between the two methods of MLS estimation comes from lesser reliability of the first method under conditions when accidental deviations become significant, i.e., if the number of mice is low. However, the first method is important here, since for the experimental group no curve fit could be done, and only the first method could be used. Therefore, to compensate for the low number at the end of the control study (two mice), we correct this by averaging the values obtained from the two methods of MLS determination:

$$\begin{array}{l} {\text{MLS}}^{C}=\frac{\Delta_{\text{2}}\cdot {\text{MLS}}_{\text{1}}^{C}+\Delta_{\text{1}}\cdot {\text{MLS}}_{2}^{C}}{\left(\Delta_{\text{1}}+\Delta_{\text{2}}\right)}=17,4\pm 0.5\text{ months}, \end{array}$$

where Δ_1_ and Δ_2_ are standard deviations of ${\text{MLS}}_{1}^{C}$ and ${\text{MLS}}_{2}^{C}$, respectively.

The initial size of the experimental group, which underwent transplantation, was 56 mice. By the age of 15 months (the beginning of injections of donor BM), 31 mice survived. Nine mice died during the injection on the operating table (embolism), 5 more mice were sacrificed for tissue sampling, and the rest (42 mice) died naturally (Figure 1; Table 1S). The immediate death of 9 embolic mice out of ~150 injections might be caused by researcher inaccuracy in eliminating aggregates from injected material as well as by injection-induced platelet activation in the bloodstream. An additional control conducted on 15 old animals of a similar breed who received a total of 75 injections of 300 μl of HBSS-heparin showed no lethality. Thus, the embolic risk requires further investigation. It is possible that the most senile individuals died after BM injections. Taken this consideration into account we decided to include embolic mice in statistic analysis to calculate MLS. In statistic analysis the embolic mice were treated as if they died from natural causes. The mortality dynamics in the experimental group is shown as open circles in Figure 1, and the stepped curve reflects the dynamics of mortality corrected for the 9 embolic animals. The 5 sacrificed mice were excluded from all graphs and the statistics.

For the calculation of MLS after BM transplantations, we used the maximal ages of 10% of the most long-lived mice from the experimental group, which correspond to ages of the 5 last mice: 21.0, 22.5, 22.7, 23.8, and 24.2 months, which gives MLS^*exp*^ = 22.8 ± 0.8 month.

Thus, the nonablative syngeneic BM transplantation increased the maximum lifespan of the mice by 100% × (22.8–17.4)/17.4 = 31 ± 5%, and the survival time from the beginning of transplantation increased (22.8–15)/(17.4–15) = 3.25 ± 0.3-fold.

At the age 19.3 months, when the last mouse of the control group died sedentary, almost immobile, and hunchback with poor hair, the transplanted mice were active, had an even spine, and shiny even hair (Videos S1A,B of control and experimental mice at age 19.3 months are presented in the Supplemental Material).

**Figure 2:**
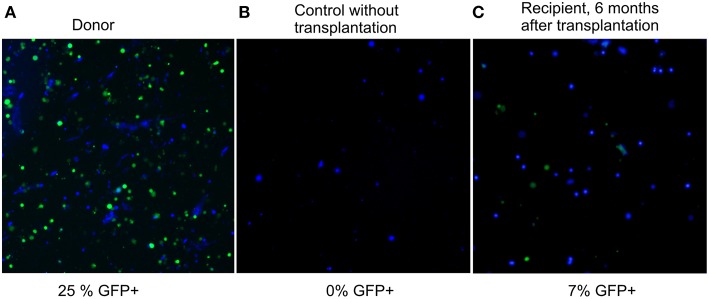
Fluorescence images of preparations of bone marrow suspension of donor, control, and transplanted mice. Overlay of fluorescence microphotographs at 380 nm (nuclei stained by NucBlue, shown in blue here), and 485 nm (GFP^+^, shown in green). Cellinsight CX7, magnification 200X. **(A)** donor BM, the content of nucleated GFP^+^ cells is 25%; **(B)** BM of a control GFP-mouse without transplantation, the content of nucleated GFP^+^ cells is 0%; **(C)** BM of a recipient 6 months after transplantation, the content of nucleated GFP^+^ cells is 7%.

Six months after the transplantation, GFP-fluorescence was detected in 7% of recipient bone marrow nucleated cells (Figure 2, panel 3). Considering that only a quarter of nucleated cells fluoresce in the BM of a heterozygous donor (25%, panel 1 of Figure 2), the degree of chimerism of the transplanted recipient was (7/25) × 100% = 28%. Thus, more than one fourth of recipient BM cells 6 months after nonablative transplantation were of donor origin; therefore, the extension of the lifespan must be a consequence of not only the paracrine effect, but also of cellular replacement. Fluorescent structures were also visualized in other tissues of the recipients, such as liver, spleen, kidneys, and muscles 6 months after the last injection (Figure S1 of Supplementary Material).

## Discussion

In this study, a significant (30%) increase in maximum lifespan of mice was found after nonablative transplantation of 100 million nucleated bone marrow (BM) cells from young donors, initiated at the age that is equivalent to 75 years for humans. Moreover, rejuvenation was accompanied by a high degree of BM chimerism for the nonablative approach. Six months after the transplantation, 28% of recipients' BM cells were of donor origin. The degree of chimerism obtained in the Quesenberry laboratory ranged from 17.5 to 15.5% per 100 million injected cells [7% per 40 million BM cells injected or 31% per 200 million BM cells injected (Colvin et al., 2004)]. This is about half the integration efficiency observed in our work (28% per 100 million BM cells injected). The relatively high chimerism efficiency that we found is most likely due to the advanced age of our recipients having a depleted BM pool, in contrast to the young nonablative recipients in the work by the cited authors.

In addition to the higher incorporation rates, there are more reasons why the nonablative setting is preferable for old recipients. These are lesser risks of infections and of graft-vs.-host disease, threatening to ablated patients, while graft rejection by nonablated recipients is less probable in the elderly than at a younger age because of naturally weaker immune system in the elderly. Even in the absence of histocompatibility, when allogeneic BM was used in a nonablative experiment instead of syngeneic BM, no lifespan shortening of the experimental group was observed (Karnaukhov et al., 2015). Obviously, at an old age the immune system is already too passive to reject donor BM, but it still efficiently suppresses infection and graft-vs.-host reaction, which makes it unnecessary and undesirable to use ablative conditioning in the elderly.

The advantages of lowly ablative transplantation in older age groups were shown for hematooncologic human patients older than 50 years: the 95% survival line decreased 5 months after a myeloablative transplantation, while 95% of nonmyeloablative patients were alive for at least 13 months; both 1 and 2 year survival rates were 50% higher in the nonmyeloablative than in myeloablative cohorts over 50 years of age (Alyea et al., 2005). Nonablative or lowly ablative transplantation of SCs has already proved its usefulness in clinical practice for some human pathologies: multiple sclerosis, blood oncology, hereditary diseases (Mielcarek et al., 2003; Alyea et al., 2006).

On the bases of the above and our data, we advocate a more rapid implementation of nonablative SC transplantation into the clinic not only for pathology treatment, but also for rejuvenation. The efficiency of SC transplantation can be further increased by optimizing the methods of preparation, storage, conditioning, and selection of SCs, in particular, a significant increase in SC homing was found during antiapoptotic treatment (Kollek et al., 2017). Cultured mesenchymal cells were shown to effectively prolong the life of myeloablated mice and reduce their osteoporosis (Shen et al., 2011); human mesenchymal SCs transplanted to rats extended their lifespan and increased their stamina and memory (Dajeong et al., 2015). Good effect in elderly patients was demonstrated recently with allogeneic mesenchymal stem cells, and a clinical trial has started (Tompkins et al., 2017). The richest source of highly proliferative mesenchymal stem cells is menstrual blood (Kovina et al., 2018). Since not only freshly isolated but also cryopreserved SCs could be effective, the cryobanking of bone marrow SCs, cord blood SCs, and menstrual SCs would eliminate the shortage of histocompatible donor SCs. In the light of the above, the present work on rejuvenation potential of stem cell therapy, as well as other studies that compare effectiveness of different stem cells, donor age, and isolation and storage methods are deemed to be important for the development of antiaging therapy.

## Conclusions

1. For the first time rejuvenation therapy was started so late, at the point when half of the animals had already died, and the high (31 ± 5%) extension in maximal lifespan of the remaining animals was found. such significant effect on the maximal lifespan, unlike the median lifespan fluctuations, indicates that BM transplantation affects the intrinsic aging mechanism. The life-extending effect was significantly stronger than in earlier works with similar design (no irradiation or chemotherapy, no hereditary pathologies in recipients, advanced age at the start of the BM administration) because of (i) the larger amount of transplanted material and (ii) the close relation of the donors and recipients. The result is encouraging for clinical adaptation for aged humans (70–80-years old).
2. The observed lifespan extension was accompanied by extension of an active and healthy life period.
3. The bone marrow chimerism of recipients after BM transplantation was significant (28% of nucleated BM cells were of donor origin) and permanent (it lasted for at least 6 months after the transplantation), indicating that rejuvenation is caused not only by the paracrine effect but also by direct cell replacement.

## Ethics Statement

The animal study was carried out in accordance with the recommendations of the local ethical committee of Sechenov First Moscow State Medical University and the study protocol was approved by the local ethical committee of Sechenov First Moscow State Medical University (protocol 05-14).

## Author Contributions

MVK: idea and coordination of work; AVK: mathematical analysis and statistical treatment of the data; MEK, ALK, SG, LS, and YK: bone marrow isolation and intravenous injections; YK and MVK: analyzed the data and wrote the manuscript; EB: veterinary work; EK: preparing illustrations; MB: bone marrow staining and chimerism analysis; TD and AL: administrative support and discussion.

### Conflict of Interest Statement

The authors declare that the research was conducted in the absence of any commercial or financial relationships that could be construed as a potential conflict of interest.

## Supplementary Material

Figure S1 Fluorescence and phase-contrast photographs of tissues of recipient mice 1–6 months after the transplantation are given in the overlay. Fluorescent cells of donor origin are present. Upper panel: from left to right: spleen x200, kidney x200 (2 photos). Lower panel: liver x400, liver x200 and liver of the non-transplanted control x200.

Video S1 **(A)** – the last control mouse at the age of 19.3 months, 1 day before natural death. **(B)** – mice of the treated group of the same age of 19.3 months.

Table 1S Life span and cause of death of mice from control and experimental groups. Before age of 15, 5 months for the control group and 13 months for the experimental group the age of death was not monitored regularly. From this moment onwards the mice were monitored twice a week. For the calculation of MLS after BM transplantations, we used the maximal ages of 10% of the most long-lived mice from the experimental group, which correspond to ages of the 5 last remaining mice: 21.0, 22.5, 22.7, 23.8, and 24.2 months.
