# Supplementary figures and images for: Extension of Maximal Lifespan and High Bone Marrow Chimerism After Nonmyeloablative Syngeneic Transplantation of Bone Marrow From Young to Old Mice
Source: Front Genet. 2019 Apr 12;10:310. doi: 10.3389/fgene.2019.00310 (PMC6473025; doi:10.3389/fgene.2019.00310)

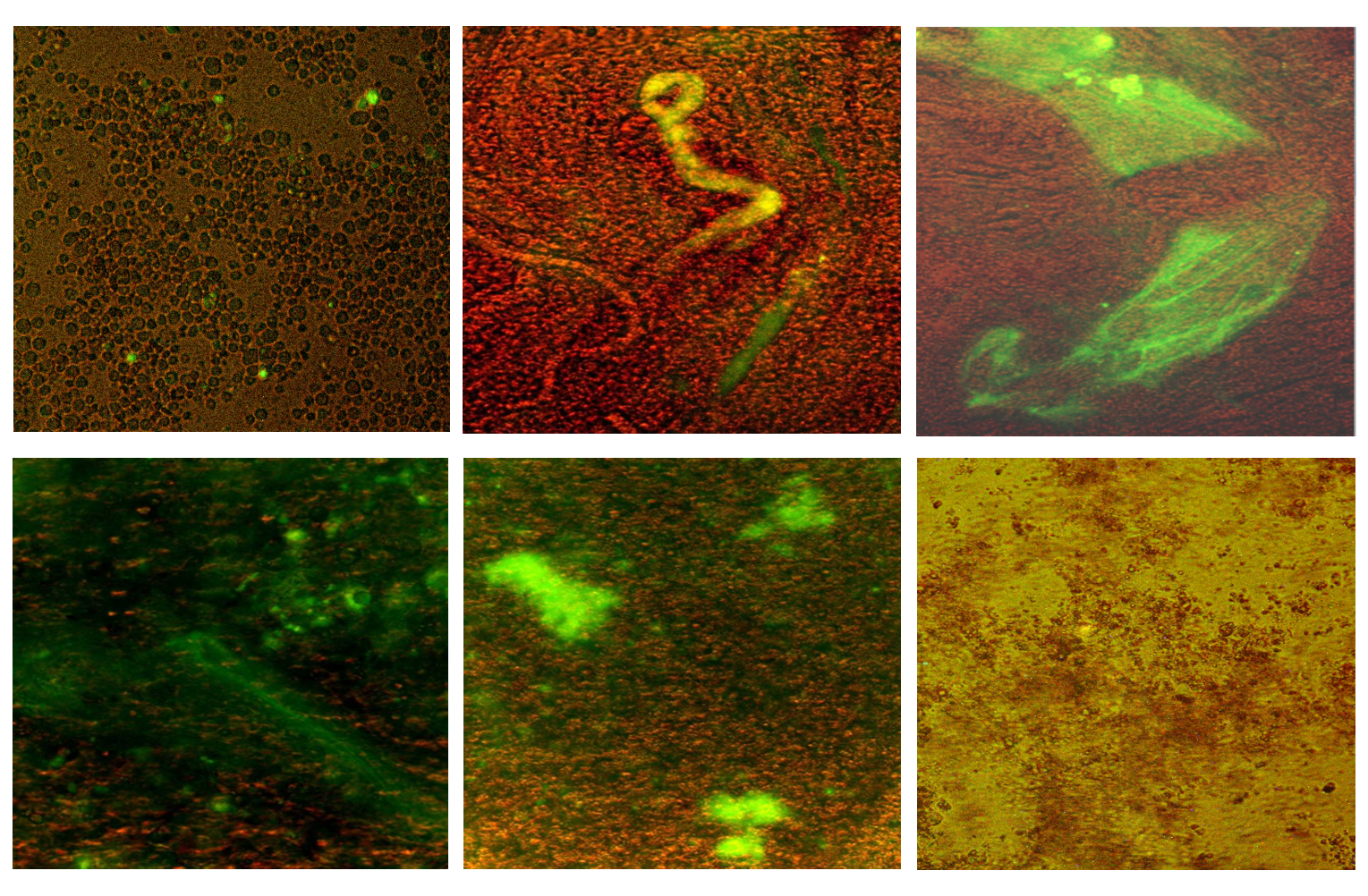

Supplement: Figure S1 — Fluorescence and phase-contrast photographs of tissues of recipient mice 1–6 months after the transplantation are given in the overlay. Fluorescent cells of donor origin are present. Upper panel: from left to right: spleen x200, kidney x200 (2 photos). Lower panel: liver x400, liver x200 and liver of the non-transplanted control x200. [file Image_1.TIF]
