# Supplementary material for: Extension of Maximal Lifespan and High Bone Marrow Chimerism After Nonmyeloablative Syngeneic Transplantation of Bone Marrow From Young to Old Mice
Source: Front Genet. 2019 Apr 12;10:310. doi: 10.3389/fgene.2019.00310 (PMC6473025; doi:10.3389/fgene.2019.00310)
Supplement: Table 1S — Life span and cause of death of mice from control and experimental groups. Before age of 15, 5 months for the control group and 13 months for the experimental group the age of death was not monitored regularly. From this moment onwards the mice were monitored twice a week. For the calculation of MLS after BM transplantations, we used the maximal ages of 10% of the most long-lived mice from the experimental group, which correspond to ages of the 5 last remaining mice: 21.0, 22.5, 22.7, 23.8, and 24.2 months. [file Table_1.doc]

| mouse NN | Life span (months) | Cause of death |
| --- | --- | --- |
| **Control group** | |  |
| 1-15 | less than 15.5 months | natural |
| 16 | 16.1 | natural |
| 17 | 16.2 | natural |
| 18 | 16.6 | natural |
| 19 | 17.6 | natural |
| 20 | 19.3 | natural |
| **Experimental group** | |  |
| mouse NN | Life span (months) | Cause of death |
| 1-8 | less than 13 months | natural |
| 9 | 13.2 | natural |
| 10 | 13.2 | natural |
| 11 | 13.5 | natural |
| 12 | 13.7 | natural |
| 13 | 13.8 | natural |
| 14 | 14.0 | natural |
| 15 | 14.0 | natural |
| 16 | 14.0 | natural |
| 17 | 14.1 | natural |
| 18 | 14.1 | natural |
| 19 | 14.1 | natural |
| 20 | 14.5 | natural |
| 21 | 14.6 | natural |
| 22 | 14.6 | natural |
| 23 | 14.7 | natural |
| 24 | 14.7 | natural |
| 25 | 14.8 | natural |
| 26 | 14.9 | natural |
| Start of transplantations | |  |
| 27 | 15.1 | natural |
| 28 | 15.2 | natural |
| 29 | 15.3 | natural |
| 30 | 15.3 | natural |
| 31 | 15.4 | natural |
| 32 | 15.4 | died during transplantation |
| 33 | 15.4 | died during transplantation |
| 34 | 15.4 | died during transplantation |
| 35 | 15.4 | died during transplantation |
| 36 | 15.4 | died during transplantation |
| 37 | 16.0 | died during transplantation |
| 38 | 16.0 | died during transplantation |
| 39 | 16.2 | died during transplantation |
| 39 | 16.5 | natural |
| 40 | 16.5 | natural |
| 41 | 16.8 | died during transplantation |
| 42 | 17.1 | natural |
| 43 | 17.4 | natural |
| End of transplantations | |  |
| 44 | 18.0 | natural |
| 45 | 18.6 | natural |
| 46 | 18.6 | natural |
| 47 | 21.0 | natural |
| 48 | 22.5 | natural |
| 49 | 22.7 | natural |
| 50 | 23.8 | natural |
| 51 | 24.2 | natural |

**Table 1S**. Life span and cause of death of mice from control and experimental groups. Before the age of 15.5 months for control group and 13 months for experimental group the age of death was not monitored regularly. From this moment onwards the mice were monitored twice a week. For the calculation of MLS after BM transplantations, we used the maximal ages of 10% of the most-long-lived mice from the experimental group, which correspond to ages of the 5 last remaining mice: 21.0, 22.5, 22.7, 23.8, and 24.2 months.
